# Formation of multinucleated giant cells and microglial degeneration in rats expressing a mutant Cu/Zn superoxide dismutase gene

**DOI:** 10.1186/1742-2094-4-9

**Published:** 2007-02-28

**Authors:** Sarah E Fendrick, Qing-Shan Xue, Wolfgang J Streit

**Affiliations:** 1 Department of Neuroscience, University of Florida College of Medicine and McKnight Brain Institute, 100 Newell Drive, Gainesville FL 32611, USA

## Abstract

**Background:**

Microglial neuroinflammation is thought to play a role in the pathogenesis of amyotrophic lateral sclerosis (ALS). The purpose of this study was to provide a histopathological evaluation of the microglial neuroinflammatory response in a rodent model of ALS, the SOD1^G93A ^transgenic rat.

**Methods:**

Multiple levels of the CNS from spinal cord to cerebral cortex were studied in SOD1^G93A ^transgenic rats during three stages of natural disease progression, including presymptomatic, early symptomatic (onset), and late symptomatic (end stage), using immuno- and lectin histochemical markers for microglia, such as OX-42, OX-6, and *Griffonia simplicifolia *isolectin B4.

**Results:**

Our studies revealed abnormal aggregates of microglia forming in the spinal cord as early as the presymptomatic stage. During the symptomatic stages there was prominent formation of multinucleated giant cells through fusion of microglial cells in the spinal cord, brainstem, and red nucleus of the midbrain. Other brain regions, including substantia nigra, cranial nerve nuclei, hippocampus and cortex showed normal appearing microglia. In animals during end stage disease at 4–5 months of age virtually all microglia in the spinal cord gray matter showed extensive fragmentation of their cytoplasm (cytorrhexis), indicative of widespread microglial degeneration. Few microglia exhibiting nuclear fragmentation (karyorrhexis) indicative of apoptosis were identified at any stage.

**Conclusion:**

The current findings demonstrate the occurrence of severe abnormalities in microglia, such as cell fusions and cytorrhexis, which may be the result of expression of mutant SOD1 in these cells. The microglial changes observed are different from those that accompany normal microglial activation, and they demonstrate that aberrant activation and degeneration of microglia is part of the pathogenesis of motor neuron disease.

## Background

Amyotrophic lateral sclerosis (ALS) is an adult onset neurodegenerative disease characterized by selective loss of upper and lower motor neurons. Loss of motor neurons results in muscle paralysis and ultimately death due to respiratory failure. 5–10% of ALS cases are familial inherited in an autosomal dominant pattern, and of familial ALS cases 20% have been linked to mutations located in the Cu/Zn superoxide dismutase 1 (SOD1) gene [1-4]. The discovery that SOD1 gene mutations are linked to motor neuron disease has facilitated development of transgenic rodent models to mimic human disease [1,2,5], and these have provided important leads towards understanding the molecular pathology of ALS. Since SOD1 is critically involved in eliminating superoxide, an undesirable byproduct of oxidative phosphorylation and a potential source of oxidative damage, the fact that transgenic animals with SOD1 mutations show unchanged or even elevated SOD1 activity has led to the conclusion that it is not a lack of enzymatic activity that contributes to disease development but rather some acquired toxic property of the enzyme [6,7]. Thus the question arises, what are the cellular targets of this toxicity? Several studies have shown that expression of mutant SOD1 limited to motor neurons is insufficient to cause motor neuron degeneration [8,9], and work by Cleveland and co-workers has generated findings, which show that toxicity to motor neurons requires damage from mutant SOD1 acting within nonneuronal cells [10] and, more specifically, that microglial cells are important for late stage disease development [11]. These findings point towards a critical involvement of microglia in motor neuron disease development, yet the nature of microglial-neuronal interactions that lead to motor neuron degeneration remains unknown. One possibility, which has also been studied extensively in the context of other neurodegenerative diseases, notably Alzheimer's disease, is the notion of chronic and detrimental microglial neuroinflammation [12]. According to this theory, activated microglia are seen as the main cellular source of inflammatory mediators in the CNS and as such are thought to be potentially neurotoxic [13,14]. Chronic neuroinflammation is thought to be involved also in the pathogenesis of ALS based on a variety of *in vivo *and *in vitro *studies concerned with studying microglial activation using both human and animal tissues [15-20].

In order to learn more about the role of microglia in the pathogenesis of motor neuron disease, we set out to investigate microglial activation in the G93A SOD1 mutant rat during natural disease progression. The results reported here are unexpected in that they reveal a highly abnormal microglial reaction that does not meet the criteria of an anticipated, characteristic neuroinflammatory response.

## Methods

### Animals

Animal use protocols were approved by the University of Florida Institutional Use and Care of Animals Committee (IUCAC). All transgenic animals used in this study were male Sprague Dawley NTac:SD-TgN(SOD1G93A)L26H rats obtained from Taconic Farms where animals were screened extensively for infections prior to shipping. Upon arrival animals were housed under SPF conditions. Age-matched, wild type Sprague Dawley rats were purchased from Harlan. The time course of disease progression varied among individual animals, but in general once symptoms developed disease progression was quite rapid causing death of most animals by 5 months of age.

To examine microglial morphology, microglial markers were used at three stages of the disease: 1) presymptomatic stage, where animals had no apparent muscle weakness. Animals studied in this group were aged 74–84 days; 2) early symptomatic stage (onset), where animals first showed evidence of hind limb weakness. Animals studied in this group were aged 113–117 days; 3) late symptomatic (end stage), where animals were no longer able to right themselves after 30s. Animals studied in this group were aged 135–156 days. For each of the three disease stages, 4 transgenic and 4 age-matched wild type control animals were used.

### Tissue processing and immunohistochemistry

Animals were deeply anesthetized with pentobarbital and perfused transcardially with phosphate buffer saline (PBS) followed by a fixative solution containing 4% paraformaldehyde in PBS. The spinal cord and brain were dissected out and fixed overnight in 4% paraformaldehyde at 4°C, transferred to 30% sucrose and then frozen. Lumbar spinal cord, cortical, and brainstem sections were cut in the coronal plane at 20 μm on a cryostat, mounted on slides and air dried. Sections were pretreated in PBS with 0.5% Triton X-100 for 15 min, blocked in 10% normal goat serum for 30 min and incubated overnight at room temperature in the primary antibody diluted in buffer. The primary antibodies included MRC OX-42 (Serotec, Cambridge, UK) and MRC OX-6 (Serotec, Cambridge, UK) at 1:500. The slides were rinsed in PBS and incubated in secondary antibody (1:500) for 1 h. Following incubation, slides were rinsed and Horseradish Peroxidase Avidin D was applied (1:500; Vector, Burlingame, CA) and incubated for 30 min. Slides were washed and immunoreactivity was visualized with 3,3'-diaminobenzidine (DAB)-H_2_O_2 _substrate. After a brief rinse, slides were dehydrated in increasing concentrations of ethanols, cleared in xylene, and coverslipped using Permount mounting medium (Fisher Scientific).

OX-42 immunoreactivity in the ventral spinal cord was quantified using Image Pro Plus software (version 4.5.1, Media Cybernetics, Carlsbad, CA). The area occupied by stained cells was highlighted and measured for each section of spinal cord (6 sections per animal) then expressed as a percentage of total area of ventral spinal cord. Using GraphPad Prism software (San Diego, CA) a t-test was performed to determine statistical significance between transgenic SOD 1 and control animals at each time point. A one-way ANOVA was performed to compare differences among the transgenic animals followed by a Tukey multiple comparison test.

### Paraffin processing and lectin histochemistry

Animals were deeply anesthetized and transcardially perfused with phosphate buffer saline (PBS) followed by a fixative solution containing 4% paraformaldehyde. The spinal cord and brain were dissected out and fixed 2 h in 4% paraformaldehyde. The tissue was dehydrated through ascending alcohols, cleared in xylenes and embedded in paraffin. Serial 7 μm coronal sections were collected and mounted on slides. Sections were deparaffinized through xylenes, graded alcohols and rinsed in PBS. Next, the slides were trypsin treated (0.1% trypsin, 0.1% CaCl_2_) for 12 min at 37°C. Following a 10 min wash the slides were incubated overnight at 4°C in lectin GSA I-B_4_-HRP (Sigma Chemical Co.) diluted 1:10 in PBS containing cations (0.1 mM of CaCl_2_, MgCl_2 _and MnCl_2_) and 0.1% Triton X-100. After overnight incubation slides were briefly rinsed in PBS and visualized with 3,3'-diabimobenzidine (DAB)- H_2_O_2 _substrate. Sections were counterstained with cresyl violet, dehydrated through ascending alcohols, cleared in xylenes and coverslipped with Permount.

## Results

### Development of microgliosis during natural disease progression in the spinal cord

**Figure 1:**
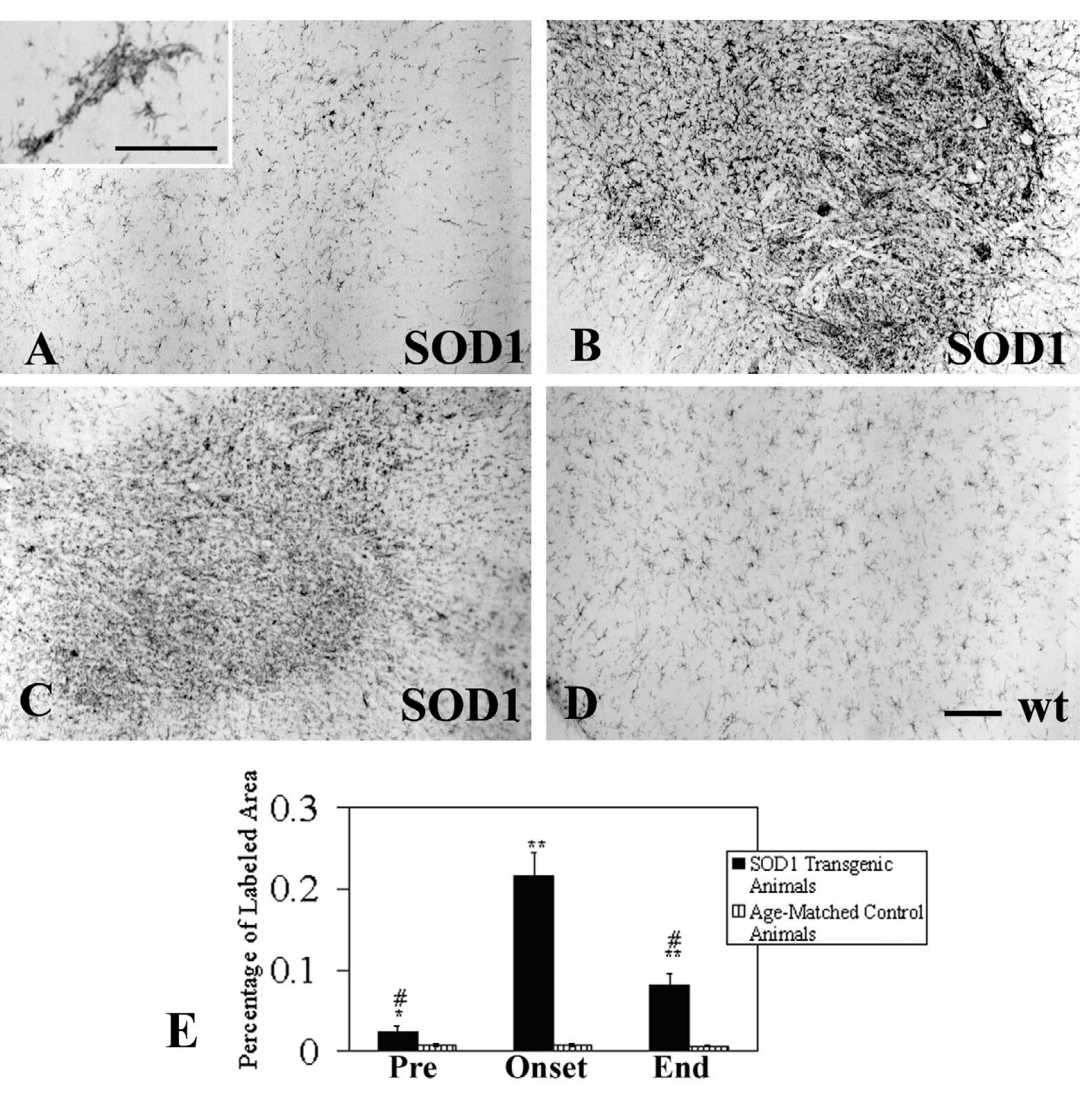
Microglial staining with OX-42 immunohistochemistry in the spinal cord during three different stages of motor neuron disease progression. **A**, presymptomatic stage; inset shows early microglial fusion in spinal cord. **B**, disease onset; **C**, end stage; **D**, wild type control. Note the dramatic increase in microglial staining with OX-42 during onset (**B**) and its subsequent decline during end stage (**C**). Scale bar: 200 μm. **E**, morphometric quantification of microglial immunostaining with OX-42 during disease development; * p < 0.05 and ** p < 0.001 with respect to age-matched controls; # p < 0.05 with respect to onset group.

The CR3 complement receptor recognized by OX-42 antibody is expressed constitutively by all resting and activated microglial cells [21]. OX-42 immunoreactivity observed in presymptomatic SOD1 transgenic rats was similar to that seen in wild type control, i.e. there was uniform staining of all resting microglia (Figs. 1A,D). Occasionally, in these presymptomatic animals cell fusions involving several microglia were observed (Fig. 1A, inset). The onset of symptoms was associated with a dramatic increase in OX-42 staining in the ventral horn due to much greater microglial cell numbers (Fig. 1B). Many of these seemingly activated microglia were clustered and/or fused into multi-cellular aggregates. In end stage animals, overall immunoreactivity with OX-42 was decreased compared to that seen in animals with disease onset (Fig. 1C). This unexpected diminution in microglial staining was due to widespread degenerative cytoplasmic fragmentation affecting most, if not all microglia within the ventral horn (see below). The qualitatively evident increases and decreases in immunoreactivity were confirmed through quantitative morphometric measurements (Fig. 1E).

**Figure 2:**
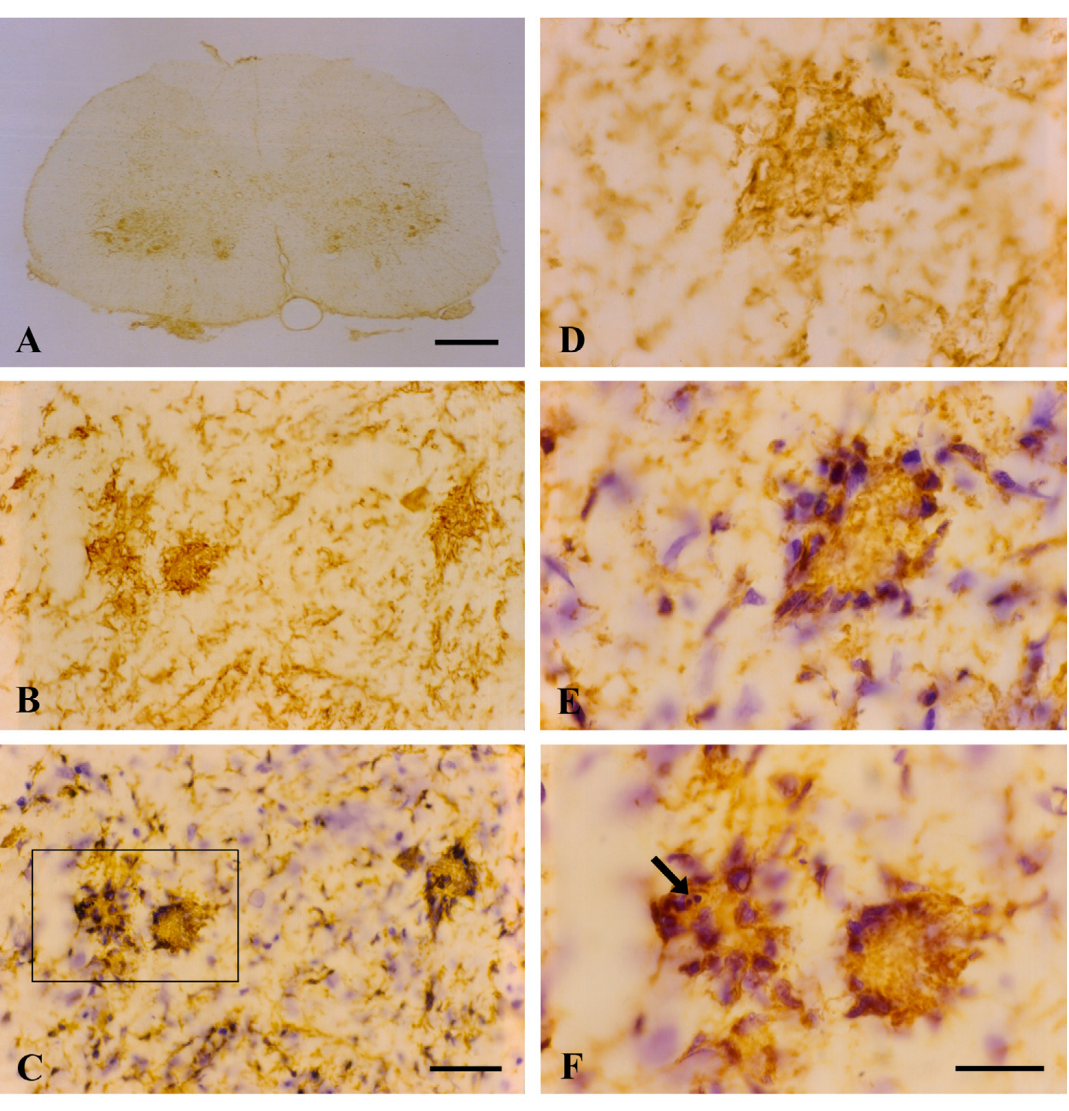
OX-42 immunohistochemistry during symptomatic phase of disease. **A**, low power view reveals intensified immunoreactivity in spinal cord ventral horns; multiple large, rounded spots are visible. **B**, higher power view of large immunoreactive spots is suggestive of phagocytic clusters. **C**, same field as in B; counterstaining with cresyl violet facilitates identification of large immunoreactive spots as multinucleated giant cells. **D**, **E**, the same microscopic field prior to and after cresyl violet counterstaining reveals a well-formed multinucleated giant cell of the Langhans type. **F**, enlargement of framed area in C shows apoptotic microglial nucleus (arrow) within a giant cell. Scale bars: 500 μm (A), 40 μm (B, C), 20 μm (D-F).

**Figure 3:**
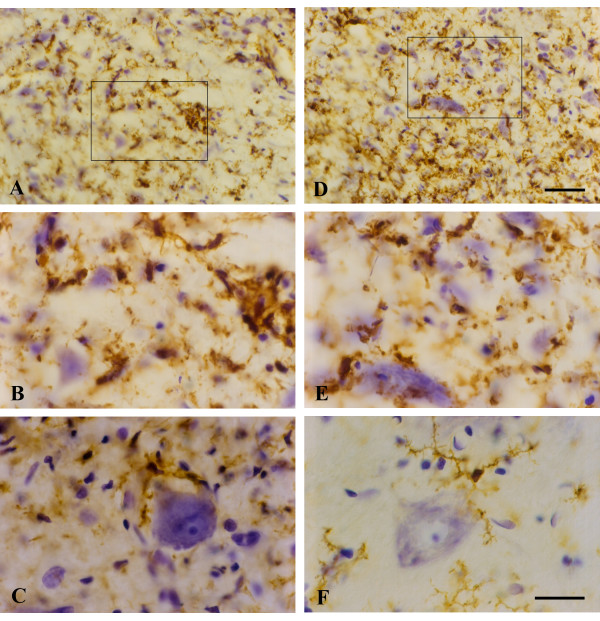
OX-42 immunohistochemistry during end stage disease demonstrates extensive microglial cytoplasmic fragmentation (**A-E**). **A**, **D**, two different views of spinal ventral gray matter demonstrate loss of microglial cell integrity and widespread punctate staining indicative of cytorrhexis. Note that many neurons remain stained with cresyl violet. **B**, enlargement of framed area in A shows detail of microglial cytorrhexis, including a disintegrating giant cell on the right. **E**, enlargement of framed area in D shows detail of microglial cytorrhexis. **C**, motor neuron in SOD1^G93A ^rat reveals intense hyperchromasia with cresyl violet and nuclear cap. **F**, normal motor neuron and microglia from wild type spinal cord. Scale bars: 40 μm (A, D); 20 μm (B, C, E, F).

With onset of symptoms, there was apparent activation of microglia as judged by the dramatic increase in OX-42 immunoreactivity in the spinal gray matter. Examining sections at low power clearly revealed pronounced spots of enhanced OX-42 staining in the ventral horns (Fig. 2A), and these were judged initially to be due to the formation of microglial phagocytic clusters around dying motor neurons, as this would be a normal response to motor neuron death. However, when spots of intense OX-42 immunoreactivity were examined at higher power (Fig. 2B,D) they appeared unusual in that individual microglial phagocytes were not discernable. Subsequent counterstaining of these sections with cresyl violet allowed us to conclude that the OX-42 reactive structures were, in fact, not phagocytic clusters but represented multinucleated giant cells (Figs. 2C,E). These giant cells were found in all SOD1^G93A ^transgenic rats studied. They formed apparently as a result of multiple microglial cells fusing together into sizable syncytia (40–50 μm) that often showed a circular arrangement of microglial nuclei about their periphery (Fig. 2E). This kind of nuclear arrangement is classically associated with multinucleated giant cells of the Langhans type. The cytoplasmic interior of Langhans giant cells appeared granular and fragmented, suggesting ongoing deterioration. A few of the giant cells revealed the presence of apoptotic bodies, evident as nuclear fragments (Fig. 2F), but overall apoptotic bodies either inside or outside of giant cells were sparse. Microglia dispersed in between giant cells revealed relatively normal process-bearing morphology and lacked the conspicuous hypertrophy that is characteristic of activated microglia. (Fig. 2E). However, some sections showed ongoing microglial cytorrhexis, i.e. fragmentation of the cytoplasm. Cytorrhexis became conspicuous in animals that were in the terminal stages of the disease process (Fig. 3) and was evident as a loss of discernable microglial cell structure and presence of abundant OX-42 immunoreactive fragments of microglial cytoplasm dispersed throughout the spinal gray matter (Figs. 3A,B,D,E). Occasional giant cells could still be observed during end stage disease, however, most of these showed signs of deterioration evident by increased irregularity of their shape and nuclear arrangement, as well as by increased granularity and fragmentation (Figs. 3A,B). In some sections, neurons remained stained with cresyl violet suggesting residual preservation of neuronal integrity. However, pathological features were evident in motor neurons, including most notably intense hyperchromia with formation of a nuclear cap consisting of condensed chromatin material (Fig. 3C). This neuronal appearance stood in stark contrast to that of normal motor neurons as seen in wild type animals (Fig. 3F).

### Histopathology in the brain stem

**Figure 4:**
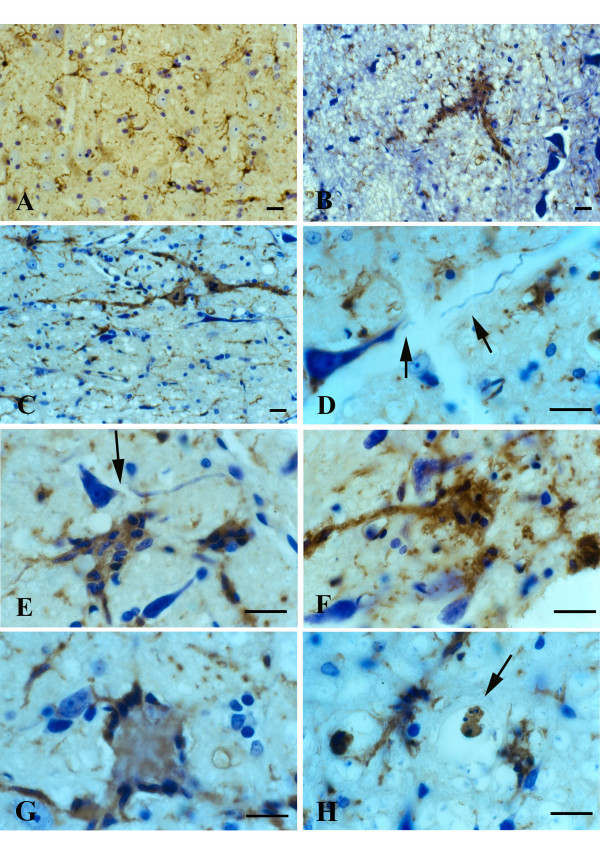
Lectin staining of microglia in the brainstem (level of cranial nerve VII) in wildtype animals (**A**) and in late symptomatic/end stage animals (**B-H**). Cresyl violet counterstain. **A**, microglia show normal ramified morphology. **B**, a large lectin-positive aggregate of fused microglia is evident in severely vacuolated brainstem tissue. Note enlarged perineuronal spaces to the right. **C**, string-like microglial fusions extend over long distances. **D**, breakage of neuronal process, probably a dendrite, from cell body within markedly vacuolated space (arrows). **E**, two multinucleated microglial giant cells are seen below a neuron with broken off process (arrow). **F**, large multinucleated giant cell displaying vacuolization is present amidst numerous microglial cytoplasmic fragments. **G**, multinucleated giant cell of the Langhans type displaying characteristic peripheral arrangement of nuclei. **H**, rounded lectin-positive microglial cell (arrow) within vacuolated space displays nuclear fragmentation indicative of apoptosis. Scale bars: 20 μm (A-H).

Sections from the brainstem at the level of cranial nerve VII during disease onset and end stage were marked by changes indicative of severe neuropathology (Fig. 4). They included prominent, widespread vacuolization of the extracellular space and hyperchromia of neuronal processes. Often neurites appeared physically separated (as if torn) from neuronal cell bodies leaving one or more distinct stumps on the perikaryon (Fig. 4D,E). The changes affecting microglia were striking in that multinucleated giant cells were present throughout any given section. These consisted of fused microglial cells that gave rise to a variety of bizarrely shaped cellular fusions which, in some cases, extended for more than one hundred micrometers in length (Figs. 4B,C,F). Microglial fusions varied in size, sometimes involving only a few cells, and other times twenty or more. Although not obviously associated with vascular channels, some microglial giant cells due to their elongated shape seemed to have formed along blood vessels (Fig. 4C). Presence of giant cells was observed in all animals regardless of whether they were at an early or late symptomatic stage of motor neuron disease. They were scattered seemingly at random throughout the brainstem and not limited to any particular nucleus or tract, and often displayed the classic morphological features of Langhans type giant cells (Fig. 4G).

Within vacuolated spaces rounded, shrunken microglia exhibiting nuclear fragmentation or shrinkage (pyknosis) were identified using lectin histochemical staining (Figs. 4H).

### Microglia in midbrain and cerebral cortex

**Figure 5:**
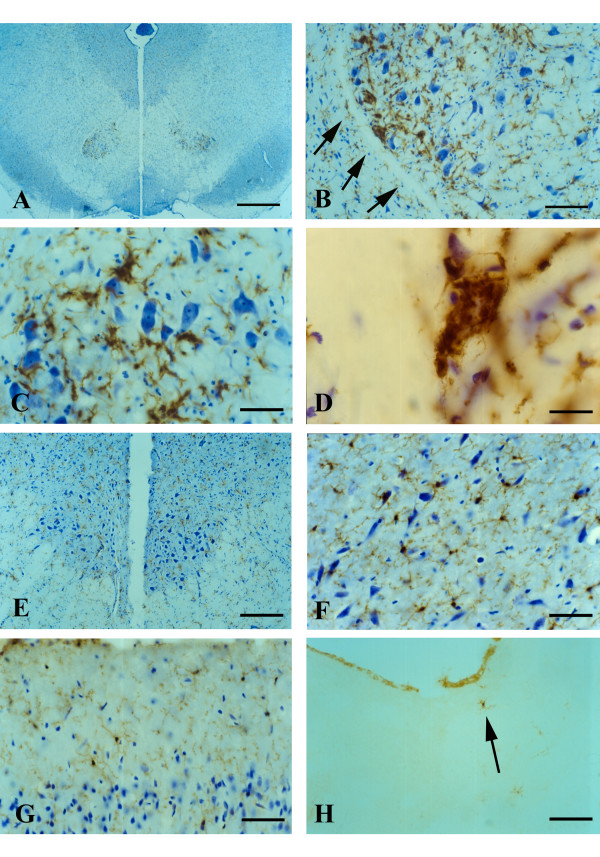
Visualization of microglia in midbrain with GSA-I-B_4 _lectin (**A-F**) and in motor cortex with OX-42 (**G**) and OX-6 (**H**) during symptomatic disease. **A**, low power view of midbrain reveals enhanced lectin staining in the red nucleus. **B**, higher magnification shows that enhanced lectin reactivity is confined strictly to red nucleus region (arrows indicate perimeter of red nucleus). **C**, microglial fusions are interspersed with rubrospinal neurons that appear undamaged. **D**, lectin-positive microglial fusion (giant cell) within red nucleus. **E**, oculomotor nucleus reveals normal-appearing motor neurons and lack of microgliosis. **F**, substantia nigra (pars compacta) shows presence of normal, ramified microglial cells. **G**, motor cortex shows normal, ramified microglia. **H**, single, ramified microglial cell positive with OX-6 (arrow) near lateral ventricle. Scale bars: 400 μm (A); 200 μm (E); 100 μm (B,H); 50 μm (C,F,G); 20 μm (D).

Microglial fusions similar to those seen in the spinal cord and brainstem level were found also in the red nucleus of the midbrain (Figs. 5A–D). The specificity with which these microglial fusions were restricted to the red nucleus area was remarkable, as they were visible even at the lowest magnification (Fig. 5A). Microglia outside of the red nucleus displayed normal, ramified morphology. Rubrospinal neurons appeared normal in size and morphology, as well as in number, and there was no evidence to suggest that any of these neurons were undergoing degeneration. Rubrospinal neurons were not encircled by activated microglia. It is noteworthy also that motor neurons in the oculomotor nucleus, which appears with the red nucleus in the same sections, revealed no evidence of degenerative changes, and microglia here were normal and non-activated in appearance. Similarly, microglia in the substantia nigra appeared completely normal (Fig. 5F). Somewhat surprisingly, we also found no evidence at all for microglial activation or abnormalities in the motor cortex of animals, regardless of disease stage, with any of the microglial markers employed (Figs. 5G,H).

## Discussion

**Figure 6:**
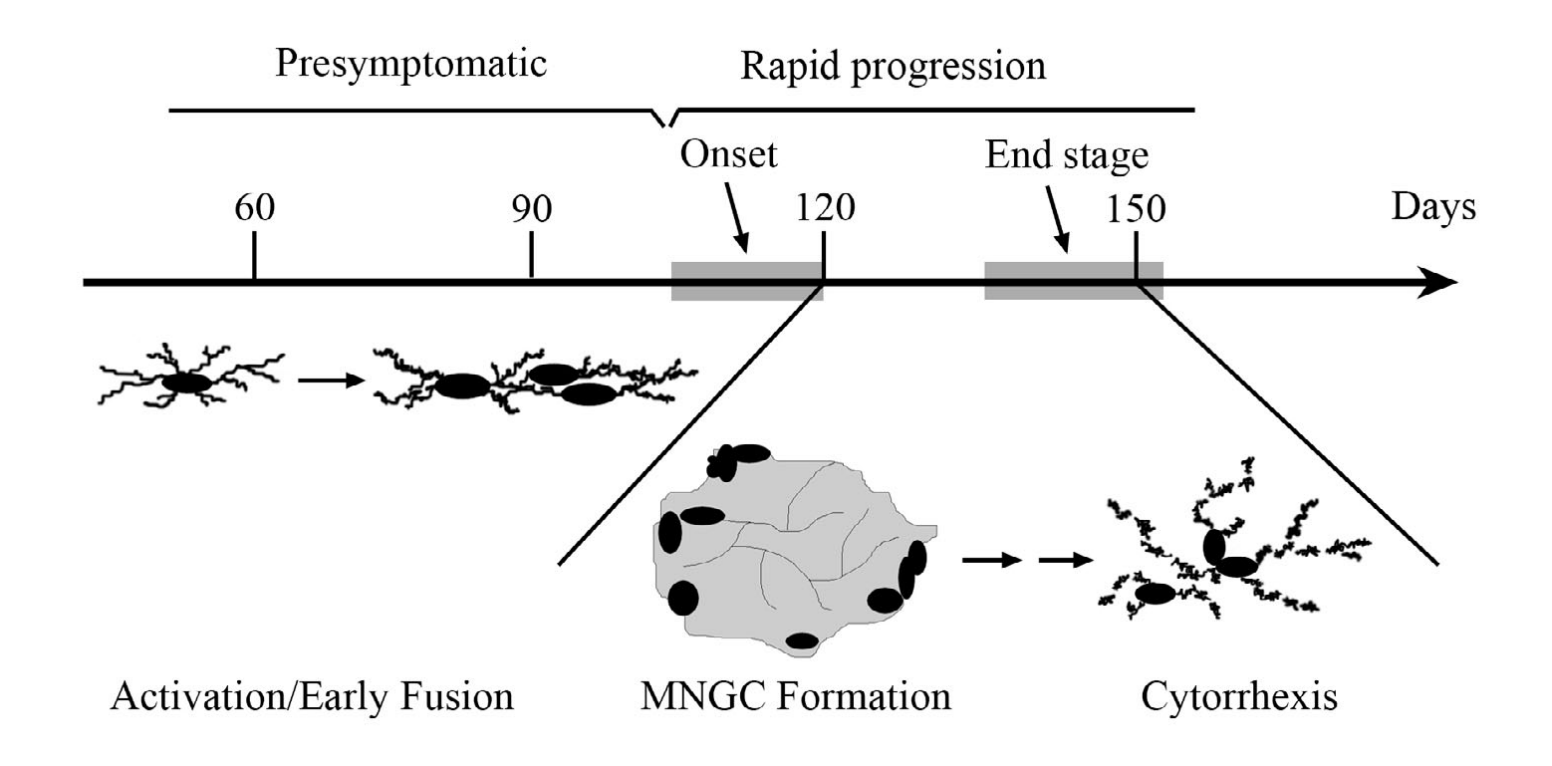
Schematic depicting the approximate time course of motor neuron disease development and the accompanying microglial changes in SOD1^G93A ^rats. Note that disease onset and subsequent development of end stage disease is variable among individual animals.

The purpose of the current study was to perform an investigation of microgliosis in a recently developed rat model of ALS involving expression of a mutated human SOD1 transgene (G93A) [5]. Although these animals, similar to their murine counterparts, reportedly mimic many of the histopathological features of human ALS, including glial activation [5,19], until now a detailed analysis of reactive microgliosis has not been performed. Our current results show that the microgliosis that occurs in SOD1^G93A ^rats is atypical and marked by some highly unusual features in microglial cells that are indicative of cellular dysfunction. The key microglial aberrations found consist of fusion into giant cells and cytorrhexis (Fig. 6). These features are not observed normally during microglial activation and they lead us to conclude that this particular animal model of ALS is characterized by microglial degeneration rather than by microglial neuroinflammation. It is therefore conceivable that neurodegeneration occurs as a consequence of glial cell deterioration.

Prior work in ALS rodent models involving SOD1 mutations has generated clues about an involvement of glial cells. Damage to astrocytes has been described to occur concomitant with degeneration of motor neurons prompting the hypothesis that astrocytic damage promotes motor neuron degeneration [22]. However, subsequent experiments showed that restricted expression of mutant SOD1 genes in astrocytes is not sufficient to cause motor neuron degeneration [23]. Notwithstanding these findings, more recently it was determined using chimeric animals consisting of mixtures of normal cells and cell expressing human mutant SOD1 that nonneuronal cells containing mutant SOD1 are indeed required to cause damage to motor neurons, whereas wildtype nonneuronal cells promote motor neuron survival [10,24]. In addition, recent work has shown that mutant SOD1 acting within microglial cells specifically is a primary determinant of late stage disease progression [11]. These observations gain added significance when considered together with the current findings showing widespread microglial degeneration in the spinal cord gray matter of end stage animals, because it now seems clear that mutant SOD1 is particularly toxic to microglia and that SOD1-mediated microglial degeneration is linked to a terminal neurodegenerative disease state. Thus, loss of microglial cells could be very detrimental to neuronal survival [25]. Future research may be directed towards elucidating the molecular mechanisms that underlie SOD1's selective microglial toxicity, and towards ways of inhibiting it as a strategy for new ALS treatments.

We use the term "cytorrhexis" to describe the kind of microglial degeneration we observed in SOD1^G93A ^rats because it involves disintegration of the cell's cytoplasm rather than of its nucleus. Cytorrhexis has been used previously only to describe neuronal necrosis resulting from excitotoxicity [26], but extending its use to describe microglial cytoplasmic deterioration is appropriate since this form of cell death does not involve the nuclear disintegration (karyorrhexis) that is characteristic of apoptosis. Cytorrhexis therefore describes accidental, rather than programmed, microglial cell death. Our inability to detect large numbers of apoptotic microglia in the tissues studied indirectly supports the idea that cytorrhexis is the "preferred" mode of microglial cell death during the toxic disease state thought to be generated by mutant SOD1 expression. Finding widespread microglial degeneration in this particular animal model of neurodegenerative disease strongly supports the broader concept that microglial abnormalities characterize other neurodegenerative conditions as well [27-29].

Perhaps the earliest sign of an aberrant microglial response in SOD1 mutant rats is reflected in our observation of occasional microglial fusions in presymptomatic animals. We suspect that with disease onset these progress to produce the conspicuous multinucleated giant cells composed of many microglia fused into large syncytia. The occurrence of microglial giant cells throughout the lumbar spinal gray matter, as well as the brainstem, and especially their selective localization in the red nucleus, raises the intriguing possibility that their formation is related to the fact that these regions all give rise to fibers that project onto ventral motor neurons. It is conceivable therefore that a signal is transmitted retrogradely from ventral horn cells to these supraspinal regions to trigger formation of microglial fusions, consistent with the notion of disease spread from an initially affected region [11]. However, at the same time the notable absence of microglial abnormalities and/or activation in the motor cortex reported here would argue against this idea. Additional studies providing more detailed mapping of the location of giant cells could be helpful in this regard.

Fusion of microglia into giant cells represents an anomalous type of cellular behavior, since microglia are normally "territorial" and exhibit strong contact inhibition. Microglial giant cells have never been described to occur *in situ *in rat brain, but they can form spontaneously *in vitro *using cultured microglia from a variety of species [30-33]. Multinucleated giant cells are a pathological hallmark in human brain during infectious diseases, most notably in HIV/AIDS encephalopathy [34,35], and since microglia are the main cellular target of HIV-1 in the brain it is thought that presence of virus within microglia causes the cells to fuse with each other. However, the exact mechanisms that produce microglial fusions in the SOD1^G93A ^rat are unknown and require additional studies. But regardless of the mechanism(s) involved, it seems clear from the current observations that fusion of microglia into giant cells is an abnormal cellular response that can progress further to produce frank microglial degeneration evident as cytorrhexis. It is unknown currently whether microglial cytorrhexis occurs in HIV encephalopathy, but the fact that neurodegenerative changes accompany advanced, untreated HIV encephalopathy (clinically evident as AIDS-dementia complex) raises the intriguing possibility that microglial degeneration may be part of the pathogenesis of cognitive dysfunction associated with HIV encephalitis. In addition, descriptions of an ALS-like syndrome in HIV-infected subjects [36-38] leads us to hypothesize that dysfunctional or degenerating microglia may be a common denominator in these two seemingly unrelated conditions.

The thought that microglial neuroinflammation promotes motor neuron degeneration can be traced back to the initial descriptions of activated microglia in human ALS tissues [16]. Since then, the idea of detrimental neuroinflammation has received additional support from a variety of studies documenting microglial activation and increased production of proinflammatory substances in human ALS tissues, serum and CSF as well as in SOD1 transgenic mice [15,20,39-44]. With regard to the SOD1 transgenic rat, the only report thus far concerning neuroinflammation has described elevated gene expression of proinflammatory mediators in the spinal cord, as well as downregulation of the neuroprotective factor VEGF [45].

## Conclusion

Our current findings provide a perspective on the pathogenesis of neurodegenerative disease that is different from the neuroinflammation theory which claims that chronic neuroinflammation leads to motor neuron degeneration [14]. We propose that, rather than being overly activated, the brain's immune cells fail in performing normal, neuroprotective functions and that degeneration of microglia contributes to neurodegeneration. This may significantly change future approaches towards treatment by pharmacological and other means.

## Competing interests

The author(s) declare that they have no competing interests.

## Authors' contributions

SF carried out the histopathological studies and drafted the manuscript. QX participated in analysis of histopathological findings and design of figures and schematics. WS conceived and directed the study, and completed the final version of the manuscript.
